# Die Universitätsmedizin in Bayern im Rahmen der COVID-19-Pandemie

**DOI:** 10.1007/s00063-021-00793-5

**Published:** 2021-03-01

**Authors:** M. Dommasch, M. Zimmermann, K.-G. Kanz, C. D. Spinner

**Affiliations:** 1 Fakultät für Medizin, Zentrale Interdisziplinäre Notaufnahme, Klinikum rechts der Isar, Technische Universität München, Ismaninger Str. 22, 81675 München, Deutschland; 2 Fakultät für Medizin, Zentrale Notaufnahme, Universitätsklinikum Regensburg, Universität Regensburg, Regensburg, Deutschland; 3 Fakultät für Medizin, Klinik und Poliklinik für Innere Medizin II, Klinikum rechts der Isar, Technische Universität München, München, Deutschland

**Keywords:** SARS-CoV‑2, COVID-19, Pandemieplanung, Versorgungsstrategie, Intensivmedizin, SARS-CoV‑2, COVID-19, Pandemic preparation, Care strategy, Intensive care

## Abstract

**Hintergrund:**

Anfang 2020 wurde deutschlandweit das Gesundheitswesen bedingt durch die coronavirus disease 2019 (COVID-19)-Pandemie auf einen Notbetrieb umgestellt. In Bayern wurde durch das zuständige Innen- und Gesundheitsministerium zu Beginn der ersten Welle eine Allgemeinverfügung erlassen, in der unter anderem die Organisation der Krankenhausbelegung, Neukonzeption der Informationstechnologie(IT)-Steuerung und Meldepflichten angeordnet wurden. Ziel dieser Auswertung war es, die Bedeutung der universitären Medizin für die stationäre Behandlung von COVID-19-Patienten in Bayern zu untersuchen.

**Methoden:**

Es erfolgte eine retrospektive Auswertung aller stationär behandelten COVID-19-Patienten, die über das Modul IVENA Sonderlage (IVENA eHealth, [IVENA, interdisziplinärer Versorgungsnachweis, mainis IT-Service GmbH, Offenbach am Main, Deutschland]) gemeldet wurden. Hierbei wurden die gemeldeten Behandlungstage aller bayerischen Kliniken, die an der Versorgung von COVID-19-Patienten teilgenommen haben, ausgewertet.

**Ergebnisse:**

Im Rahmen der ersten Welle der COVID-19-Pandemie wurden 90,9 % der Behandlungstage von kommunalen und öffentlichen sowie privaten Krankenhäusern in Bayern bereitgestellt. Neben der medizinischen Versorgung von COVID-19-Patienten mit komplexen Verläufen (20 % der Intensivstations[ICU]- und Intermediate-care-Stations [IMC]-Behandlungstage) leistete die Universitätsmedizin in Bayern mit ihren Kliniken einen relevanten wissenschaftlichen Beitrag und war wesentlich an der Beratung von Ärzten, Krankenhäusern und Politik zur Pandemie beteiligt.

## Abstract (en)

**Background:**

In early 2020 the German healthcare system was put into a state of emergency due to the coronavirus disease 2019 (COVID-19) pandemic. Bavaria had to deal with more severe acute respiratory syndrome coronavirus 2 (SARS-CoV-2) infections than any other German state during the first wave and currently has over 270,000 cases, accounting for about one fifth of all COVID-19 cases in Germany. The Bavarian Ministry of Interior together with the Bavarian Sate Ministry of Health and Care issued a general ruling at the beginning of the first wave that ordered the centralised organisation of hospital capacity, a redesign of the information technology (IT) management system and introduced reporting obligations for SARS-CoV-2/COVID-19. The goal of this analysis was to investigate the role that university hospitals played in the inpatient treatment of COVID-19 patients.

**Methods:**

A retrospective evaluation of all inpatient COVID-19 cases that were reported through the “IVENA Sonderlage” (Ivena eHEALTH, [IVENA, interdisziplinärer Versorgungsnachweis, mainis IT-Service GmbH, Offenbach am Main, Germany]), a special module for the “Interdisciplinary Medical Care Capacity Management System” designed for extraordinary events and circumstances, was conducted by analysing the number of reported treatment days of all Bavarian hospitals that participated in the treatment of COVID-19 patients.

**Results:**

During the first wave university hospitals provided relevant scientific contributions and played an important role in advising physicians, hospitals and politicians on the pandemic. In all, 20% of intensive care unit (ICU) and intermediate care (IMC) treatment days were provided by the university hospitals in particular for treatment of complex courses of COVID-19.

## Hintergrund

Das neuartige Severe Acute Respiratory Syndrome Coronavirus 2 (SARS-CoV-2) hat Anfang 2020 eine weltweite Pandemie ausgelöst. Bis Mitte Dezember 2020 gab es über 74 Mio. Infizierte und 1,6 Mio. Verstorbene weltweit [1]. Zur Kontrolle der coronavirus disease 2019(COVID-19)-Pandemie wurden zahlreiche Interventionen bis hin zum globalen „Lockdown“ erforderlich: So wurde auch in Bayern durch das zuständige Staatsministerium des Innern, für Sport und Integration und für Gesundheit und Pflege zu Beginn der ersten Welle am 24.03.2020 eine Allgemeinverfügung erlassen, in der unter anderem die Organisation der Krankenhausbelegung, Neukonzeption der Informationstechnologie(IT)-Steuerung und Meldepflichten angeordnet wurden. Es wurde für jeden der 26 bayerischen Zweckverbände für Rettungsdienst und Feuerwehralarmierung (ZRF) die Funktion eines Ärztlichen Leiters Führungsgruppe Katastrophenschutz (Ärztlicher Leiter FüGK) eingerichtet. Die Fallzahlen und Belegungsdaten wurden ab dem 01.04.2020 mittels eines webbasierten IT-Systems (IVENA eHealth [IVENA, interdisziplinärer Versorgungsnachweis]) verbindlich und fortlaufend erfasst [2]. Zudem wurden alle bayerischen Krankenhäuser verpflichtet, entsprechende Kapazitäten zur Behandlung von COVID-19-Patienten auszubauen. Auch in Deutschland hat der Verband der Universitätsklinika Deutschlands (VUD) in einer Pressemitteilung vom 14.03.2020 zugesichert, insbesondere Intensivkapazitäten für COVID-19-Patienten bereitzustellen [3]. Aufgrund der initial schwer einzuschätzenden Situation wurde eine sehr hohe Klinikkapazität bereitgestellt, wobei die Rolle der unterschiedlichen Versorgungsstufen der Krankenhäuser in der Pandemie initial unklar war.

Ziel dieser Auswertung war es, die Bedeutung der universitären Medizin für die stationäre Behandlung von COVID-19-Patienten deskriptiv in Bayern zu untersuchen. Hierbei ist der Freistaat Bayern das Bundesland mit einer der höchsten SARS-CoV-2-Fallzahlen der ersten Welle und hat mit derzeit mehr als 270.000 Infizierten etwa ein Fünftel aller COVID-19-Fälle in der Bundesrepublik zu verzeichnen [4].

## Methode

Ausgewertet wurden die stationär behandelten COVID-19-Patienten aller bayerischen Kliniken, die über das Modul IVENA Sonderlage (IVENA eHealth [IVENA, interdisziplinärer Versorgungsnachweis, mainis IT-Service GmbH, Offenbach am Main, Deutschland]) gemeldet wurden [5]. Die retrospektive Analyse umfasste den Zeitraum vom 01.04.2020 bis zum 30.06.2020 (2. Quartal 2020). Es wurden die täglich gemeldete Anzahl der mit COVID-19-Patienten belegten Betten auf Normalstation (NC), Intermediate-care-Station (IMC) und Intensivstation (ICU) aller bayerischen Krankenhäuser ausgewertet.

Von den 478 bayerischen Kliniken, die im IVENA-Sonderlagen-Modul hinterlegt sind, waren 292 Kliniken an der Behandlung von COVID-19-Patienten beteiligt. Unter diesen waren auch alle 6 bayerischen Universitätskliniken. Hierzu zählen die Universitätskliniken Augsburg, Erlangen, Regensburg und Würzburg, der Universität München und der Technischen Universität München.

## Ergebnisse

**Figure 1:**
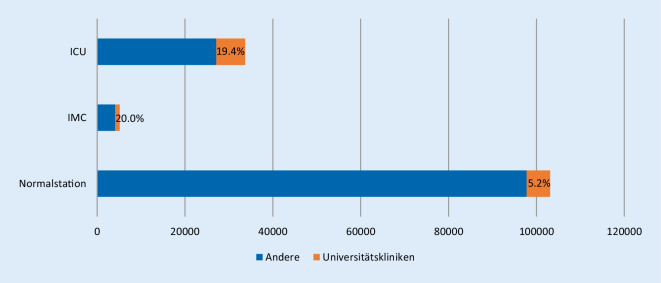

Im beobachteten Zeitraum wurden in den bayerischen Kliniken summiert 141.966 Krankenhausbetttage mit COVID-19 Patienten belegt. Der größte Teil mit 103.124 (72,6 %) Tagen wurde auf Normalstationsbetten verbraucht. Für die intensivmedizinische Behandlung von COVID-19-Patienten wurden 33.690 (23,7 %) Behandlungstage benötigt (Abb. 1). Im Monat April war der Bedarf mit über 60 % der benötigten Betten am höchsten und ist in den Folgemonaten Mai und Juni deutlich abgesunken. Im Juni betrug der Anteil nur noch 7,3 % der im beobachteten Zeitraum erhobenen Gesamtsumme an Krankenhausbetttagen.

**Figure 2:**
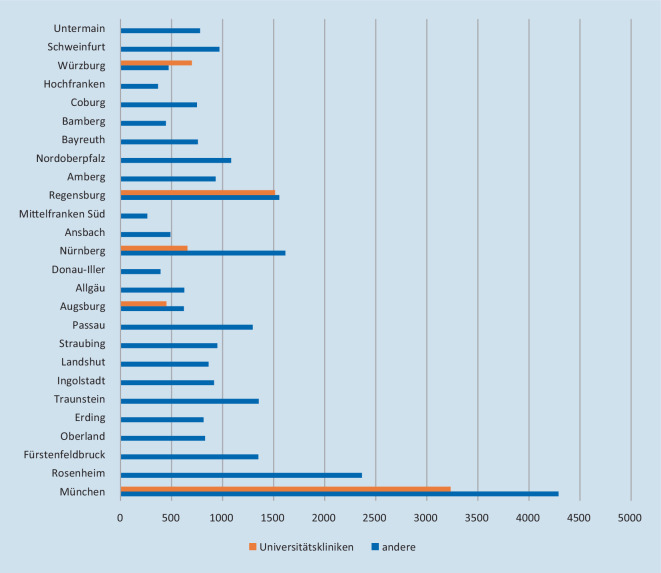

Der Großteil der Patienten wurde in den freigemeinnützigen, öffentlich-rechtlichen und privaten Kliniken behandelt. Der Anteil an allen Behandlungstagen lag hier bei 90,9 %. Die bayerischen Universitätskliniken hatten demnach einen Anteil von 9,1 % der COVID-19-Behandlungstage. Es zeigt sich, dass der Anteil an intensivmedizinischen Betten an den Universitätskliniken deutlich überwog. Im untersuchten Zeitraum haben die bayerischen Universitätskliniken 20,0 % aller IMC- und 19,4 % aller ICU-Behandlungstage abgedeckt (Abb. 1). In den einzelnen ZRF, in denen die Universitätskliniken angesiedelt sind, war dieser Anteil noch höher (Abb. 2).

## Diskussion

Die vorliegende Datenanalyse zeigt, dass der mehrheitliche Teil der in Bayern stationär zu behandelnden COVID-19-Patienten in den 292 kommunalen und öffentlichen sowie privaten Krankenhäusern, die an der Versorgung dieser Patienten teilgenommen haben, stattgefunden hat. Mit einem Anteil von etwa 10 % haben die 6 bayerischen Universitätskliniken einen nicht unerheblichen Beitrag geleistet. Zudem wurden knapp 20 % der Intensivbetten und IMC-Betten für COVID-19-Behandlung von den Universitätskliniken bereitgestellt. Ursächlich für den hohen Anteil an intensivmedizinischen Behandlungen ist am ehesten die Versorgungstufe als Supramaximalversorger mit den entsprechenden umfangreichen intensivmedizinischen Ressourcen wie differenzierter Beatmungstherapie und spezialisierten Organersatzverfahren (ECMO). Die Übernahme von Patienten aus überregionalen oder internationalen Risikogebieten mit Versorgungsengpässen gehörte auch zu den Aufgaben der Universitätskliniken.

Neben der Versorgung schwerstkranker und intensivpflichtiger COVID-19-Patienten und dem Ausbau der virologischen PCR-Testungen an den universitären Standorten haben die Universitäten im Rahmen der Pandemie auch ihren wissenschaftlichen Beitrag insbesondere in den Bereichen der Notfallmedizin, Virologie, Epidemiologie und Infektiologie geleistet. Hinzu kommen die Beratung und Bereitstellung von Fachinformationen für andere Krankenhäuser, niedergelassene Kolleginnen und Kollegen sowie Gesundheitsämter und Politik.

Die vorliegende Analyse hat folgende Limitationen: Es konnten lediglich die täglich gemeldeten Betten, die von COVID-19-Patienten belegt waren, ausgewertet werden. Die Liegedauer oder der Schweregrad der Erkrankung ist aus den vorliegenden Daten nicht zu erheben, zudem handelt es sich um eine Selbstauskunft der einzelnen bayerischen Kliniken mit entsprechenden Fehlern in der Übermittlung der Daten an IVENA.

## Fazit für die Praxis

- Im Rahmen der ersten Welle der COVID-19-Pandemie wurden 90,9 % der Behandlungstage von kommunalen und öffentlichen sowie privaten Krankenhäusern in Bayern bereitgestellt.
- Etwa 20 % der ICU- und IMC-Behandlungstage wurden von den Universitätskliniken in Bayern übernommen.
- Neben der medizinischen Versorgung von COVID-19-Patienten mit komplexen Verläufen leistete die Universitätsmedizin in Bayern mit ihren Kliniken einen relevanten wissenschaftlichen Beitrag und war wesentlich an der Beratung von Ärzten, Krankenhäusern und Politik zur Pandemie beteiligt.
